# Identification and evaluation of quantitative trait loci underlying resistance to multiple HG types of soybean cyst nematode in soybean PI 437655

**DOI:** 10.1007/s00122-014-2409-5

**Published:** 2014-10-15

**Authors:** Yongqing Jiao, Tri D. Vuong, Yan Liu, Clinton Meinhardt, Yang Liu, Trupti Joshi, Perry B. Cregan, Dong Xu, J. Grover Shannon, Henry T. Nguyen

**Affiliations:** 1 Division of Plant Sciences and National Center for Soybean Biotechnology (NCSB), University of Missouri, Columbia, MO 65211 USA; 2 Department of Computer Science, Informatics Institute and Christopher S. Bond Life Sciences Center, University of Missouri, Columbia, MO 65211 USA; 3 Soybean Genomics and Improvement Laboratory, USDA-ARS, Beltsville, MD 20705 USA; 4 Division of Plant Sciences and NCSB, University of Missouri, Delta Center, P.O. Box 160, Portageville, MO 63873 USA

## Abstract

****Key message**:**

**We performed QTL analysis for SCN resistance in PI 437655 in two mapping populations, characterized CNV of** ***Rhg1*** **through whole-genome resequencing and evaluated the effects of QTL pyramiding to enhance resistance.**

**Abstract:**

Soybean cyst nematode (SCN, *Heterodera glycines* Ichinohe) is one of the most serious pests of soybean worldwide. PI 437655 has broader resistance to SCN HG types than PI 88788. The objectives of this study were to identify quantitative trait loci (QTL) underlying SCN resistance in PI 437655, and to evaluate the QTL for their contribution to SCN resistance. Two F_6:7_ recombinant inbred line populations, derived from cv. Williams 82 × PI 437655 and cv. Hutcheson × PI 437655 crosses, were evaluated for resistance to SCN HG types 1.2.5.7 (PA2), 0 (PA3), 1.3.5.6.7 (PA14), and 1.2.3.4.5.6.7 (LY2). The 1,536 SNP array was used to genotype the mapping populations and construct genetic linkage maps. Two significant QTL were consistently mapped on chromosomes (Chr.) 18 and 20 in these two populations. One QTL on Chr. 18, which corresponds to the known *Rhg1* locus, contributed resistance to SCN HG types 1.2.5.7, 0, 1.3.5.6.7, and 1.2.3.4.5.6.7 (PA2, PA3, PA14, and LY2, respectively). Copy number variation (CNV) analysis by whole-genome resequencing showed that PI 437655 and PI 88788 had similar CNV at the *Rhg1* locus. The QTL on Chr. 20 contributed resistance to SCN HG types 1.3.5.6.7 (PA14) and 1.2.3.4.5.6.7 (LY2). Evaluation of both QTL showed that pyramiding of *Rhg1* and the QTL on Chr. 20 significantly improved the resistance to SCN HG types 1.3.5.6.7 (PA14) and 1.2.3.4.5.6.7 (LY2) in both populations. Our studies provided useful information for deploying PI 437655 as a donor for SCN resistance in soybean breeding through marker-assisted selection.

**Electronic supplementary material:**

The online version of this article (doi:10.1007/s00122-014-2409-5) contains supplementary material, which is available to authorized users.

## Introduction

Soybean cyst nematode (SCN, *Heterodera glycines* Ichinohe) is one of the most important pests of soybean [*Glycine max* (L.) Merr.] worldwide. Annual yield suppression due to SCN in the United States alone was estimated at approximately $1.5 billion (Wrather and Koenning 2006).Other than rotation with non-host crops, breeding resistant cultivars is the most economical and effective means to control this pest.

Quantitative trait loci (QTL) mapping is a powerful tool to identify genomic regions responsible for expression of important agronomic traits. Once the desired QTL are mapped, molecular markers that are tightly linked to the QTL can be applied in marker-assisted breeding to improve and shorten the process of developing resistant cultivars. To date, many QTL conferring resistance to SCN in soybean have been mapped on almost all chromosomes except for Chr. 2 (D1b) (Concibido et al. 2004; Vuong et al. 2010; Winter et al. 2007; Wang et al. 2004; Kabelka et al. 2005; Guo et al. 2005, 2006; Wu et al. 2009). Among these, two QTL, *Rhg1* on Chr. 18 and *Rhg4* on Chr. 8 (Concibido et al. 2004), were commonly mapped in various sources of resistance, primarily plant introductions (PIs). Recently, these two QTL have been successfully cloned from PI 88788 and cultivar Forrest, respectively (Cook et al. 2012; Liu et al. 2012). The SCN resistance conferred by *Rhg1* in PI 88788 was found to be controlled by three genes (Cook et al. 2012). The alteration of expression level caused by copy number variation (CNV) rather than sequence mutation for these three genes explained the phenotypic differences between susceptible and resistant varieties (Cook et al. 2012). *Rhg4* encodes a serine hydroxymethyltransferase that is essential for cellular one-carbon metabolism (Liu et al. 2012). Two point mutations in Forrest altered a key regulatory property of this enzyme, which may disturb the folate homeostasis and lead to a nutritional deficiency preventing life of the nematode (Liu et al. 2012).

In addition to cultivated soybean sources, scientists have also explored wild soybean (*Glycine soja*) germplasm to identify new genes for SCN resistance. Wang et al. (2001) reported three QTL for SCN resistance from *Glycine soja* PI 468916. Winter et al. (2007) mapped three QTL for SCN resistance from *Glycine soja* PI 464925B. These novel QTL expanded sources of SCN resistance for breeding SCN-resistant soybean cultivars.

Several sources of SCN resistance, such as PI 88788, Peking, and PI 437654 have been widely used in the development of commercial SCN-resistant soybean cultivars (Concibido et al. 2004). Continuous use of the same source of SCN resistance may lead to a genetic shift of SCN populations, causing a loss of SCN resistance in soybean. It was reported that some SCN populations recovered from the field where soybean varieties with PI 88788 resistance had been constantly used were virulent on PI 88788 (Faghihi et al. 2008). Lack of genetic diversity for SCN resistance among soybean cultivars has become a concern of soybean breeders. Identifying new sources of SCN resistance is important in controlling this pest. In addition, pyramiding of different QTL for SCN resistance from different sources may provide longer protection against SCN HG type population shifts that reduce the effectiveness of genes already employed in cultivars.

PI 437655 was first reported to be resistant to SCN HG type 0 (race 3) (Anand and Gallo 1984). Then, it was found to be resistant to SCN HG types 1.2.3- and 2.5.7 (races 4 and 1) (Anand et al. 1988; Arelli et al. 1997). In an effort to find new sources of SCN resistance, we evaluated 650 exotic soybean PIs for their resistance to multiple SCN HG types in the greenhouse. We found that PI 437655 had a lower FI for all tested SCN populations and showed broader spectrum resistance to SCN HG types than PI 88788. More importantly, we determined that PI 437655 was moderately resistant to SCN isolate LY2 (HG type 1.2.3.4.5.6.7), which was virulent on PI 437654 (Donald and Young 2004). To date, no PIs, except PI 437655, were reported to be resistant to LY2. The molecular basis underlying broad-based SCN resistance in PI 437655 is unknown. The objectives of this study were to identify the QTL responsible for resistance to multiple SCN HG types in PI 437655, and to evaluate the identified QTL for their contribution to SCN resistance.

## Materials and methods

### Plant materials

Two recombinant inbred line (RILs) populations were developed using the single-seed descent method. Population 1 (Pop1) was a population of 119 F_6:7_ RILs derived from a cross of Hutcheson × PI 437655. Population 2 (Pop2) was a population of 192 F_6:7_ RILs derived from a cross of Williams 82 × PI 437655. Hutcheson and Williams 82 are two SCN susceptible cultivars (Buss et al. 1988; Bernard and Cremeens 1988). PI 437655 is a SCN-resistant plant introduction (Anand and Gallo 1984; Anand et al. 1988; Arelli et al. 1997), originating from China and preserved in the USDA Soybean Germplasm Collection Soybean. Seeds of each RIL were planted for SCN phenotyping. Genomic DNA was extracted from a pooled sample of leaves from five seedlings of each RIL following a previously described protocol (Vuong et al. 2010).

### SCN bioassays

Seven SCN isolates, HG types 2.5.7 (PA1), 1.2.5.7 (PA2), 0 (PA3), 2.5.7 (PA5), 1.3.5.6.7 (PA14), 1.2.3.4.5.6.7 (LY1), and 1.2.3.4.5.6.7 (LY2), have been maintained for more than 30 generations and are believed to be near-homogeneous (Arelli et al. 2000). LY1 and LY2 were two SCN isolates that could reproduce on PI 437654 (Donald and Young 2004). Success of the phenotyping experiments were evaluated by SCN reaction to a set of soybean indicator lines for HG type tests (Peking, PI 88788, PI 90763, PI 437654, PI 209332, PI 89772, PI 548316, and susceptible checks (cv. Lee 74 and cv. Hutcheson) (Niblack et al. 2002). The initial screening of PI 437655 and other germplasm lines was conducted with all seven SCN isolates. Four SCN isolates, HG types 1.2.5.7 (PA 2), 0 (PA 3), 1.3.5.6.7 (PA 14), and 1.2.3.4.5.6.7 (LY2), were used for the evaluation of Pop1 and Pop2.

The SCN bioassays were performed in a greenhouse at the University of Missouri–Columbia following a well-established method (Arelli et al. 1997; Niblack et al. 2009). In brief, germinated soybean seeds were transplanted into PVC tubes (100 cm^3^) (one plant per tube). The tubes were filled with steam-pasteurized sandy soil and packed into plastic containers prior to transplanting. Each container held twenty-five tubes and was suspended over water baths maintained at 27 ± 1 °C. Five plants of each indicator line and RIL were arranged in a randomized complete block design. Two days after transplanting, each plant was inoculated with 2000 ± 25 SCN eggs. Thirty days post-inoculation, nematode cysts were washed from the roots of each plant and counted using Fluorescence-Based Imaging System (Brown et al. 2010). The female index (FI %) was estimated to evaluate the response of each plant to each HG type of SCN using the following formula:

FI (%) = (Number of female cyst nematodes on a given individual/average number of female nematodes on the susceptible check) × 100.

### Statistical analysis

Female indexes (%) among RILs of two populations were tested for normality using the PROC UNIVARIATE procedure of SAS 9.3 (SAS institute, Gary, NY, USA). A broad-sense heritability was calculated following a described method (Wu et al. 2009).

### Linkage analyses and genetic mapping

The universal soybean linkage panel 1.0 (USLP 1.0) containing 1,536 SNP loci (Hyten et al. 2008) was utilized to genotype the two RIL mapping populations using the Illumina GoldenGate assay (Fan et al. 2006). These SNP loci had been mapped onto the integrated molecular genetic linkage map (Hyten et al. 2010).

Genetic linkage maps were constructed using JoinMap 4.0 (van Ooijen 2006). A likelihood of odds (LOD) threshold score of 3.0 and a maximum genetic distance of 50 cM were used for the initial linkage grouping of markers. The soybean genetic linkage groups (LGs) (Song et al. 2004) were replaced with the new assignments of corresponding chromosome numbers (Chr.) (Grant et al. 2010).

Interval mapping (IM) was initially conducted for QTL prediction. Composite interval mapping was subsequently performed using the multi-QTL method (MQM) with the program MapQTL 5.0 and the appropriate cofactor (van Ooijen 2004). A permutation test (Churchill and Doerge 1994) was performed with 1,000 runs to determine the *P* = 0.05 genome-wide significance level for declaring a QTL significant. The proportion of the phenotypic variance explained by the QTL effects was estimated at the QTL peaks. Additive (A) effects of significant QTL were estimated from an output of the program MapQTL 5.0. The program QTLNetwork 2.0 was used to predict epistatic interactions between QTL (Yang et al. 2007).

### Whole-genome sequencing and copy number variation analysis

Whole-genome sequencing of PI 437655, PI 88788, and cv. Hutcheson was conducted using Illumina technology at the Beijing Genome Institute (BGI), Shenzhen, China, following a described protocol (Xu et al. 2013). The sequencing depth for each sample was about 15× coverage. The CNV analysis was conducted using CNV-seq software (Xie and Tammi 2009).

## Results

### Evaluation of PI 437655 for SCN resistance

In comparison with PI 88788, PI 437655 had lower FI (%) for all seven SCN isolates including SCN HG types 2.5.7 (PA1), 1.2.5.7 (PA2), 0 (PA3), 2.5.7 (PA5), 1.3.5.6.7 (PA14), 1.2.3.4.5.6.7 (LY1), and 1.2.3.4.5.6.7 (LY2) (Table 1). FI (%) in PI 437655 was reduced from 42.1 in PI 88788 to 28.6 for HG type 2.5.7 (PA1), from 44.4 to 26.2 for HG type 1.2.5.7 (PA2), from 8.1 to 4.4 for HG type 0 (PA3), from 59.0 to 38.3 for HG type 2.5.7 (PA5), from 8.4 to 5.5 for HG type 1.3.5.6.7 (PA14), from 67.9 to 56.8 for HG type 1.2.3.4.5.6.7 (LY1), and from 37.1 to 23.8 for HG type 1.2.3.4.5.6.7 (LY2) (Table 1). Therefore, PI 437655 was moderately resistant or resistant to SCN HG types 2.5.7 (PA1), 1.2.5.7 (PA2), 0 (PA3), 1.3.5.6.7 (PA14), and 1.2.3.4.5.6.7 (LY2) based on resistance standards described by Schmitt and Shannon (1992). Surprisingly, PI 437655 was moderately resistant to LY2, a SCN population that no other sources had been reported to be resistant to (Donald and Young 2004).

**Table 1:** Evaluations of PI 437655 and seven indicator lines, for resistance to different HG types of soybean cyst nematode (SCN) conducted in a greenhouse of University of Missouri–Columbia, using the rating system described by Schmitt and Shannon (1992) and Niblack et al. (2009)

Soybean lines	FI (%) of SCN HG type (SCN isolate)						
	2.5.7 (PA1)	1.2.5.7 (PA2)	0 (PA3)	2.5.7 (PA5)	1.3.5.6.7 (PA14)	1.2.3.4.5.6.7 (LY1)	1.2.3.4.5.6.7 (LY2)
PI 437655	28.6 (MR)	26.2 (MR)	4.4 (R)	38.3 (MS)	5.5 (R)	56.8 (MS)	23.8 (MR)
PI 88788	42.1 (MS)	44.4 (MS)	8.1 (R)	59.0 (MS)	8.4 (R)	67.9 (S)	37.1 (MS)
PI 548402	1.4 (R)	62.0 (S)	10.6 (MR)	14.4 (MR)	80.3 (S)	89.0 (S)	53.6 (MS)
PI 090763	9.2 (R)	9.6 (R)	1.8 (R)	4.5 (R)	43.9 (MS)	84.3 (S)	54.5 (MS)
PI 437654	1.9 (R)	1.7 (R)	1.4 (R)	7.2 (R)	9.5 (R)	74.4 (S)	47.6 (MS)
PI 209332	39.1 (MS)	41.5 (MS)	6.4 (R)	49.8 (MS)	21.7 (MR)	61.2 (S)	32.3 (MS)
PI 089772	3.2 (R)	14.7 (MR)	3.5 (R)	10.0 (R)	49.9 (MS)	64.4 (S)	83.5 (S)
PI 548316	69.6 (S)	49.6 (MS)	13.8 (MR)	65.1 (S)	30.0 (MR)	94.5 (S)	40.0 (MS)

Female index (FI) (%) values are calculated from three replicates

*R* resistant, FI < 10; *MR* moderately resistant, 10 < FI < 30; *MS* moderately susceptible, 30 < FI < 60; *S* susceptible, FI > 60

### Phenotypic variation and genetic linkage analysis

**Fig. 1:**
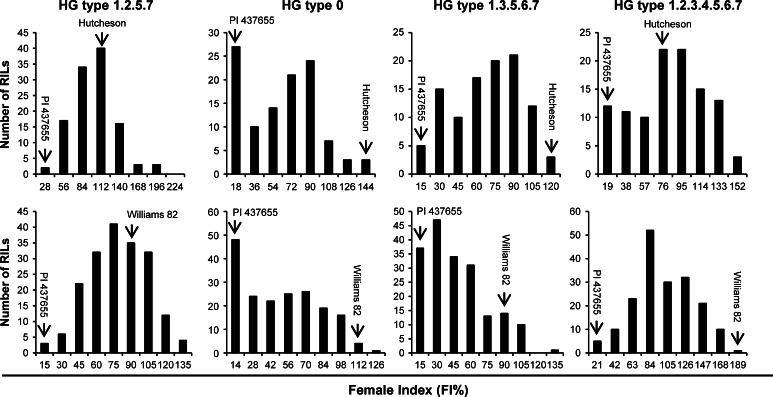
Distribution of average female index (FI %) of two F_6:7_ recombinant inbred line (RIL) populations derived from Hutcheson × PI 437655 and Williams 82 × PI 437655 crosses. Cultivars Hutcheson and Williams 82, SCN susceptible parents; PI 437655, SCN-resistant parent

The FI (%) data of Pop1 and Pop2 showed large genetic variation when assayed with each of the four SCN HG types (Table 2). The normality test by the Shapiro–Wilk (*w*) indicated that the FI data of HG types 1.2.5.7 (PA 2) and 1.2.3.4.5.6.7 (LY2) in Pop2 were normally distributed, while others were not normal (Table 2; Fig. 1). Broad-sense heritability of the FI for each HG type was calculated based upon the analysis of variance of family means. The values ranged from 0.46 to 0.68 in Pop1 and from 0.41 to 0.71 in Pop2 (Table 2).

**Table 2:** Statistics for female index (FI) (%) of two parental lines and two F_6:7_ recombinant inbred line (RIL) populations for their response to four soybean cyst nematode HG types 1.2.5.7 (PA2), 0 (PA3), 1.3.5.6.7 (PA14), and 1.2.3.4.5.6.7 (LY2), in greenhouse bioassays

HG type (SCN isolate)	Pop	FI (%)						Shapiro–Wilk (*w*)	Skew	Kurt	*H* ^2^
		P1	P2	F_6:7_ RILs families							
				Mean	Min	Max	SD				
1.2.5.7	Pop1	108.7	16.4	88.7	17.7	241.4	35.83	0.95 (0.0002)	0.98	2.63	0.57
(PA2)	Pop2	87.3	15.2	70.8	0	130.0	25.28	0.99 (0.5)	−0.18	−0.24	0.46
0	Pop1	134.0	2.4	53.3	2.6	138.6	34.18	0.95 (0.0005)	0.21	−0.73	0.71
(PA3)	Pop2	103.3	3.3	43.3	1.9	127.0	30.62	0.94 (<0.0001)	0.39	−0.92	0.66
1.3.5.6.7	Pop1	112.1	7.7	60.2	3.8	116.9	27.56	0.97 (0.04)	−0.17	−0.82	0.56
(PA14)	Pop2	81.2	2.7	39.1	0	133.0	25.92	0.94 (<0.0001)	0.80	0.11	0.68
1.2.3.4.5.6.7	Pop1	57.9	6.9	73.5	7.3	153.8	37.05	0.97 (0.008)	−0.12	−0.87	0.42
(LY2)	Pop2	172.6	17.7	89.8	0	185.2	35.76	0.99 (0.3)	0.04	−0.42	0.48

*Pop* population, *Pop1* population of 119 F_6:7_ RILs developed from a Hutcheson × PI 437655 cross, *Pop2* population of 192 F_6:7_ RILs developed from a Williams 82 × PI 437655 cross, *P1* cv. Hutcheson in *Pop1* and cv. Williams 82 in *Pop2,* *P2* PI 437655 in both populations, *SD* standard deviation, *Skew* skewness, *Kurt* kurtosis, *H* ^2^ broad-sense heritability on entry-mean basis

In Pop1, 670 markers were polymorphic between the two parents and were incorporated into linkage analysis. A linkage map spanning 3,029.50 cM across 20 chromosomes was constructed (Supplement S1). In Pop2, 768 markers were found to be polymorphic and a linkage map spanning 2,387.09 cM across 20 chromosomes was constructed (Supplement S2).

### Significant QTL for SCN resistance confirmed in the two populations

**Fig. 2:**
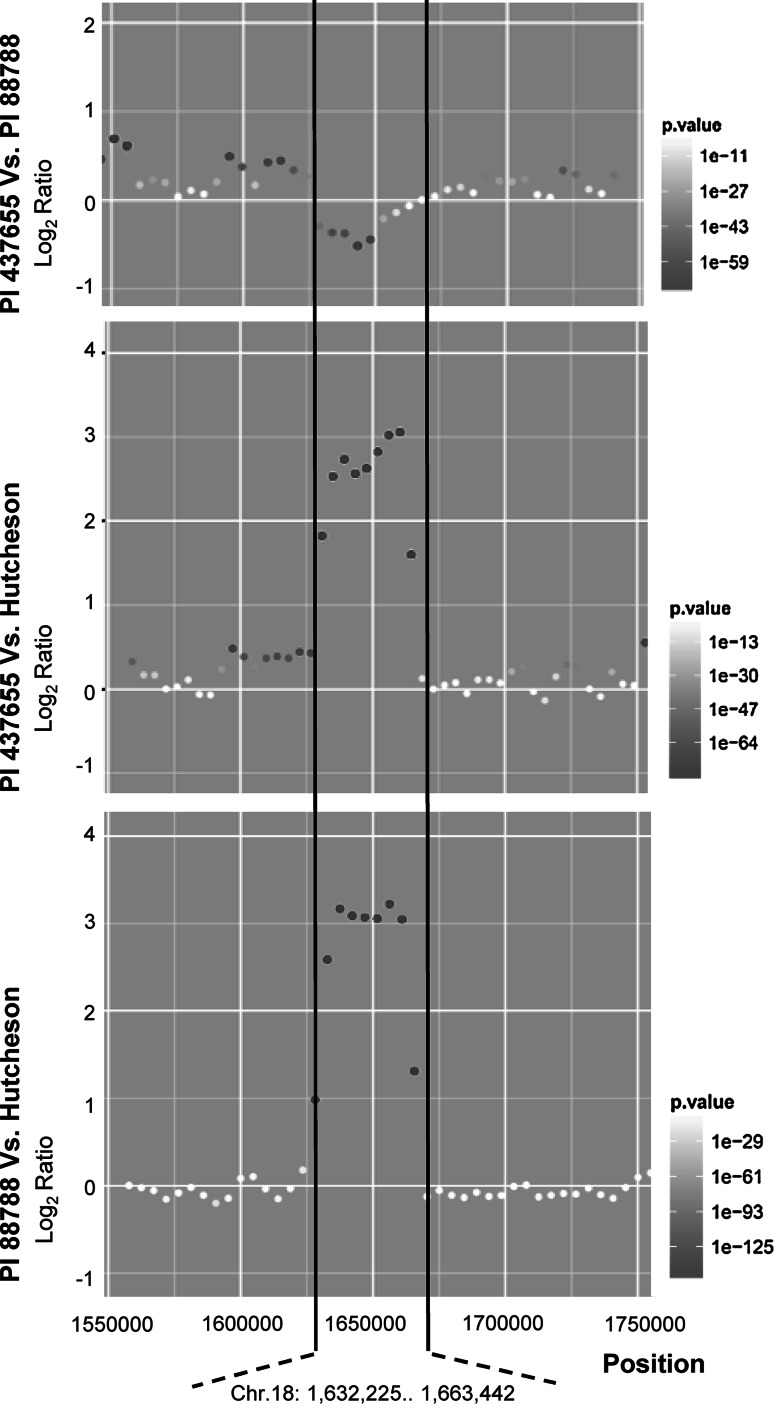
Copy number variation of the *Rhg1* locus detected by CNV-seq software among PI 437655, PI 88788, and cv. Hutcheson by use of whole-genome resequencing. The *Y* axis represents log2 ratios; the *X* axis represents genomic positions along chromosome 18 of Williams 82 reference genome. The interval between two *black lines* indicated the region of *Rhg1.* The *dot* represents the fold change of copy number variation in comparison with the corresponding soybean line. The *red color* *gradient* represents *P* value calculated on each of ratios (color figure online)

The QTL significantly associated with resistance to multi-SCN HG types 1.2.5.7 (PA2), 0 (PA3), 1.3.5.6.7 (PA14), and 1.2.3.4.5.6.7 (LY2) were detected and consistently mapped to Chr. 18 in both genetic populations (Table 3; Supplements S1 and S2). In Pop1, the total phenotypic variation explained by this QTL was 10.8 % for HG type 1.2.5.7 (PA2), 56.2 % for HG Type 0 (PA3), 51.7 % for HG Type 1.3.5.6.7 (PA14), and 30.7 % for 1.2.3.4.5.6.7 (LY2) (Table 3). In Pop2, this QTL explained 9.2 % of total phenotypic variation for HG type 1.2.5.7 (PA2), 58.1 % for HG type 0 (PA3), 27.1 % for HG type 1.3.5.6.7 (PA14), and 20.9 % for 1.2.3.4.5.6.7 (LY2) (Table 3). Although the confidence intervals mapped in the two populations were slightly different, they overlapped in the same genomic region (Table 3), indicating the same QTL was detected in both populations. The QTL on Chr. 18 has been commonly mapped in various sources, which corresponds to the known QTL *Rhg1* (Concibido et al. 2004). Moreover, previous studies showed that there are two different types of *Rhg1*: Peking-type *Rhg1* and PI 88788-type *Rhg1* (Concibido et al. 2004). PI 88788-type *Rhg1* can confer soybean SCN resistance by itself (Kim et al. 2010; Cook et al. 2012; Brucker et al. 2005). In contrast, Peking-type *Rhg1* functions only when *Rhg4* is present (Meksem et al. 2001; Brucker et al. 2005; Liu et al. 2012). In our study, a significant association was not observed between markers in the *Rhg4* interval and resistance to any SCN isolate tested (Table 3). Therefore, it was evident that PI 437655 might contain the same *Rhg1* allele as PI 88788 rather than the *Rhg1*allele from Peking. To investigate if *Rhg1* between PI 437655 and PI 88788 were the same, we conducted CNV analysis of *Rhg1* using whole-genome resequencing. Compared to the susceptible soybean cv. Hutcheson, both PI 437655 and PI 88788 had a significant increase in copy numbers of *Rhg1* (Fig. 2). No significant difference was detected for copy numbers for *Rhg1* between PI 437655 and PI 88788 (Fig. 2), which indicated that PI 437655 and PI 88788 might have the same *Rhg1* allele.

**Table 3:** Significant QTL for resistance to SCN HG types 1.2.5.7 (PA2), 0 (PA3), 1.3.5.6.7 (PA14), and 1.2.3.4.5.6.7 (LY2) identified and mapped in two recombinant inbred line (RIL) mapping populations derived from crosses of Hutcheson × PI 437655 (Pop1) and Williams 82 × PI 437655 (Pop2)

HG type (SCN isolate)	Population	QTL on Chr. (LG)	Confidence intervals	SNP closet to the peak	Peak LOD	*R* ^2^ (%)	Additive effect
1.2.5.7	Pop1	Chr.18 (G)	BARC-029369-06162–BARC-042201-08212	BARC-012289-01799	3.3	10.8	12.8
(PA2)		Chr.5 (A1)	BARC-019475-03618–BARC-053497-11882	BARC-053261-11776	3.2	10.3	11.9
	Pop2	Chr.16 (J)	BARC-042131-08181–BARC-019229-03401	BARC-030433-06867	8.1	10.1	8.3
		Chr.20 (I)	BARC-045029-08866–BARC-060361-16629	BARC-042685-08348	7.5	9.2	8.0
		Chr.18 (G)	BARC-048277-10538–BARC-014395-01348	BARC-047665-10370	7.3	9.2	8.1
		Chr.3 (N)	BARC-017957-02482–BARC-061333-17169	BARC-016199-02307	6.1	7.2	7.1
		Chr.4 (C1)	BARC-044691-08761–BARC-064861-18829	BARC-025825-05102	5.5	6.6	6.8
		Chr.15 (E)	BARC-028607-05972–BARC-058675-17461	BARC-058671-17458	4.4	5.1	5.9
0	Pop1	Chr.18 (G)	BARC-029369-06162–BARC-042201-08212	BARC-012289-01799	20.5	56.2	29.8
(PA3)		Chr.5 (A1)	BARC-050619-09775–BARC-021573-04148	BARC-019415-03923	3.3	6.6	9.7
	Pop2	Chr.18 (G)	BARC-012295-01800–BARC-048271-10520	BARC-048801-10723	45.4	58.1	24.1
		Chr.20 (I)	BARC-041155-07919–BARC-048955-10759	BARC-060361-16629	6.9	6.1	8.3
		Chr.16 (J)	BARC-042131-08181–BARC-019229-03401	BARC-030433-06867	4.3	3.4	6.0
		Chr.15 (E)	BARC-058675-17461–BARC-054023-12243	BARC-038377-10061	3.8	2.7	5.2
1.3.5.6.7	Pop1	Chr.18 (G)	BARC-029369-06162–BARC-042201-08212	BARC-012289-01799	16.3	51.7	23.0
(PA14)		Chr.20 (I)	BARC-044361-08677–BARC-059937-16229	BARC-060361-16629	4.7	12.1	10.0
	Pop2	Chr.18 (G)	BARC-012295-01800–BARC-048801-10723	BARC-012289-01799	20.0	27.1	13.9
		Chr.20 (I)	BARC-041155-07919–BARC-048955-10759	BARC-060361-16629	7.0	8.6	8.1
		Chr.16 (J)	BARC-042131-08181–BARC-019229-03401	BARC-030433-06867	6.7	7.7	7.4
		Chr.15 (E)	BARC-040185-07678–BARC-057969-15031	BARC-058493-15308	3.7	4.1	5.4
		Chr.3 (N)	BARC-053313-11792–BARC-060031-16308	BARC-057129-14594	3.6	3.9	5.3
1.2.3.4.5.6.7	Pop1	Chr.18 (G)	BARC-029369-06162–BARC-042201-08212	BARC-012289-01799	8.8	30.7	16.9
(LY2)		Chr.20 (I)	BARC-044361-08677–BARC-059937-16229	BARC-060361-16629	4.8	14.7	12.7
	Pop2	Chr.18 (G)	BARC-012295-01800–BARC-048801-10723	BARC-012289-01799	10.4	20.9	17.2
		Chr.20 (I)	BARC-044361-08677–BARC-042685-08348	BARC-045029-08866	4.8	8.9	11.1

*Pop1* population of 119 F_7_ RILs developed from a Hutcheson × PI 437655 cross. *Pop2* population of 192 F_7_ RILs developed from a Williams 82 × PI 437655 cross. The positive values for additive effects mean that SCN resistance is from PI 437655

The second QTL significantly associated with resistance to SCN HG type 1.3.5.6.7 (PA14) and 1.2.3.4.5.6.7 (LY2), were detected and consistently mapped to Chr. 20 (Table 3; Supplements S1 and S2). The total phenotypic variation explained by this QTL was 4.7 % for HG type 1.3.5.6.7 (PA14) and 4.8 % for 1.2.3.4.5.6.7 (LY2) in Pop1, and 7.0 % for HG type 1.3.5.6.7 (PA14) and 4.8 % for 1.2.3.4.5.7 (LY2) in Pop2 (Table 3). The confidence intervals mapped in the two genetic populations were similar, indicating the same QTL was detected in the two different genetic backgrounds. Previous studies showed that PI 437654, a well-known source for SCN resistance, also contained a QTL on Chr. 20, which was associated with resistance to SCN HG type 0 (PA3), 2.5.7 (PA5), and 1.3.5.6.7 (PA14) (Wu et al. 2009). The QTL detected in our study was genetically located in a genomic region similar to the one detected in PI 437654. We did not detect significant epistatic interactions between two QTL for resistance to multiple HG types on Chrs. 18 and 20 in either Pop1 or Pop2.

In addition to the QTL on Chrs. 18 and 20, several other QTL were also detected but not confirmed (Table 3; Supplements S1 and S2). In Pop1, a QTL was detected on Chr. 5 for resistance to SCN HG types 1.2.5.7 (PA2) and 0 (PA3) (Table 3; Supplement S1). In Pop2, five QTL were detected on Chrs. 16, 20, 3, 4, and 15 for resistance to HG type 1.2.5.7 (PA2); three QTL were detected on Chrs. 20, 16, and 15 for resistance to HG type 0 (PA3); and three QTL were detected on Chrs. 16, 15, and 3 for resistance to HG type 1.3.5.6.7 (PA14) (Table 3; Supplement S2). However, these QTL were not consistently mapped in both populations, which may be attributed to differences in genetic background.

### Effects of confirmed QTL to SCN resistance

We evaluated the *Rhg1* locus and the QTL on Chr. 20 for their contribution to the resistance to different SCN HG types in these two mapping populations. Our results showed that *Rhg1* significantly reduced the FI % of SCN HG types 1.2.5.7, 0, 1.3.5.6.7, and 1.2.3.4.5.6.7 (PA2, PA3, PA14, and LY2, respectively) in both populations (Table 4). Addition of the QTL on Chr. 20 in genotypes with *Rhg1* reduced FI (%) of SCN HG type 1.3.5.6.7 (PA14) and 1.2.3.4.5.6.7 (LY2) by approximately 40 % (Table 4). This indicated that pyramiding different QTL for resistance could significantly confer more stable and higher resistance in soybean to multiple HG types of SCN.

**Table 4:** Evaluation of confirmed QTL for their contribution for resistance to SCN HG types 1.2.5.7 (PA2), 0 (PA3), 1.3.5.6.7 (PA14), and 1.2.3.4.5.6.7 (LY2)

Population	Genotype		Phenotype [FI % of SCN HG type (SCN isolate)]				Number of RILs
	Rhg1	QTL on Chr. 20	1.2.5.7 (PA2)	0 (PA3)	1.3.5.6.7 (PA14)	1.2.3.4.5.6.7 (LY2)	
Pop1	−	−	104.0 ± 31.0	79.3 ± 14.0	85.5 ± 15.1	109.4 ± 26.6	13
	+	−	77.9 ± 27.3^a^	14.9 ± 6.9^a^	42.5 ± 20.5^a^	82.7 ± 21.9^a^	10
	+	+	65.0 ± 26.4^a^	9.2 ± 4.5^a,b^	24.7 ± 19^a,b^	43.3 ± 18.4^a,b^	14
Pop2	−	−	86.3 ± 23.3	75.4 ± 13.3	63.2 ± 24.6	119.9 ± 26.8	12
	+	−	68.4 ± 15.9	10.7 ± 5.2^a^	21.6 ± 4.1^a^	78.7 ± 20.4^a^	9
	+	+	52.3 ± 15.5^a^	8.3 ± 4.2^a^	11.6 ± 6.2^a,b^	49.7 ± 27.1^a,b^	7

*Pop1* population of 119 F7 RILs developed from a Hutcheson × PI 437655 cross

*Pop2* population of 192 F7 RILs developed from a Williams 82 × PI 437655 cross

− The allele from Hutcheson in Pop1 or the allele from Williams 82 in Pop2

+ The allele from PI 437655

^a^ *P* < 0.05 by a Student’s *t* test in comparison with the value of −/−

^b^ *P* < 0.05 by a Student’s *t* test in comparison with the value of +/−

## Discussion

Most QTL for SCN resistance reported in literature so far were based on a single bi-parental mapping population (Concibido et al. 2004). Although the same resistant parent was utilized to develop different mapping populations, variable QTL can be detected. For instance, in the population from a Hamilton × PI 438489B cross, Yue et al. (2001) identified QTL for SCN HG type 2.5.7 (PA5) resistance on Chrs. 11, 6, 1, and 18. However, Vuong et al. (2011) only identified a QTL on Chr. 4 for resistance to SCN HG types 2.5.7 (PA5) using the population from a Magellan × PI 438489B cross. The inconsistency of identified QTL may be attributed to the differences among genetic backgrounds.

To produce more robust QTL results, two or more mapping populations have an advantage over a single population. In our study, QTL analyses were conducted in two RIL populations derived from cv. Hutcheson crossed with PI 437655 (Pop1) and cv. Williams 82 crossed with PI 437655 (Pop2). Like the previous studies mentioned above, several QTL detected in one population were not confirmed in the other population. For instance, a QTL was mapped on Chr. 5 in Pop1 and different QTLs on Chrs. 16, 20, 3, 4, and 15 were mapped for SCN resistance in Pop2. In contrast, QTL were mapped on Chrs. 18 and 20 in both backgrounds, indicating significant QTL conferring SCN resistance from PI 437655. Apparently, these two QTL regions may be very useful as new gene sources leading to the development of new soybean varieties with improved SCN resistance.

The *Rhg1* locus has been widely mapped in various sources of SCN resistance (Concibido et al. 2004; Guo et al. 2005, 2006; Vuong et al. 2011; Wu et al. 2009). It was shown that there were two different types of *Rhg1*, PI 88788-type *Rhg1* and Peking-type *Rhg1*. These two *Rhg1* were believed to be different functional alleles (Meksem et al. 2001; Brucker et al. 2005). PI 88788-type *Rhg1* was recently cloned (Cook et al. 2012). However, the *Rhg1* allele from Peking has not been cloned. To date, many sources of SCN resistance contain *Rhg1* (Concibido et al. 2004). Because the level of SCN resistance conferred by the *Rhg1* gene was associated with CNVs (Cook et al. 2012), it is important to determine CNVs of *Rhg1* among those sources of resistance. In our study, we predicted that PI 437655 might carry PI 88788-type *Rhg1* because *Rhg4* was not detected in PI 437655. CNV analysis showed that PI 437655 had the same high number of copies of *Rhg1* as PI 88788, which indicated PI 437655 and PI 88788 might have the same level of SCN resistance or the same *Rhg1* allele.

Currently, PI 88788 has been widely employed as a predominant donor source in breeding for soybean cultivars with SCN resistance. The most important gene underlying SCN resistance in PI 88788 is *Rhg1* (Cook et al. 2012). However, several studies have reported that more and more SCN populations overcame the SCN resistance conferred by PI 88788 (Diers and Arelli 1999; Faghihi et al. 2008), which highlighted the need to develop soybean cultivars with broader and more stable resistance to multiple HG types. In addition to *Rhg1*, PI 437655 also contained a QTL on Chr. 20, not found in PI 88788 (Glover et al. 2004). This additional QTL might be the reason why PI 437655 showed better SCN resistance than PI 88788 to the four HG types evaluated in this study. RILs with the combination of the *Rhg1* locus and the QTL on Chr. 20 in each of two populations had enhanced SCN resistance than genotypes with the *Rhg1* allele alone. Thus, pyramiding different resistance QTL will likely be an effective approach to reduce the problem of increased virulence on resistance sources like PI 88788 due to genetic shifts in SCN populations. Moreover, it has been reported that there was no linkage drag for yield when either cv. Fayette or its derived cultivars were utilized in soybean breeding for Northern cultivars. The newly identified QTL in PI 437655 can be introgressed into either to cv. Fayette or its derived cultivars for broadening resistance to SCN.

### **Author contributions**

Y.J and T.D.V designed research; Y.J, T.D.V, Y.L, C.M, Y.L, T.J, P.B.C, and J.G.S performed research; Y.J analyzed data and wrote the manuscript; T.D.V, D.X, and H.T.N edited the manuscript; and H.T.N oversaw the project.

## Electronic supplementary material

Below is the link to the electronic supplementary material.

Supplementary material 1 A genetic linkage map constructed using an F_6:7_ recombinant inbred line (RIL) population from a Hutcheson × PI 437655 cross. The confidence intervals of SCN resistance QTL were shown by the bars on the right of chromosomes. The bars filled with black color represent the QTL resistant to HG type 1.2.5.7 (PA2). The bars filled no color represent the QTL resistant to HG type 0 (PA3). The bars filled with slashes represent the QTL resistant to HG type 1.3.5.6.7 (PA14). The bars filled with black dots represent the QTL resistant to HG type 1.2.3.4.5.6.7 (LY2) (TIFF 2678 kb)

Supplementary material 2 A genetic linkage map constructed using an F_6:7_ recombinant inbred line (RIL) population from a Williams 82 × PI 437655 cross. The confidence intervals of SCN resistance QTL were shown by the bars on the right of chromosome. The bars filled with black color represent the QTL resistant to HG type 1.2.5.7 (PA2). The bar filled no color represents the QTL resistant to HG type 0 (PA3). The bars filled with slashes represent the QTL resistant to HG type 1.3.5.6.7 (PA14). The bars filled with black dots represent the QTL resistant to HG type 1.2.3.4.5.6.7 (LY2) (TIFF 2817 kb)
