# Supplementary figures and images for: Identification and evaluation of quantitative trait loci underlying resistance to multiple HG types of soybean cyst nematode in soybean PI 437655
Source: Theor Appl Genet. 2014 Oct 15;128(1):15–23. doi: 10.1007/s00122-014-2409-5 (PMC4282714; doi:10.1007/s00122-014-2409-5)

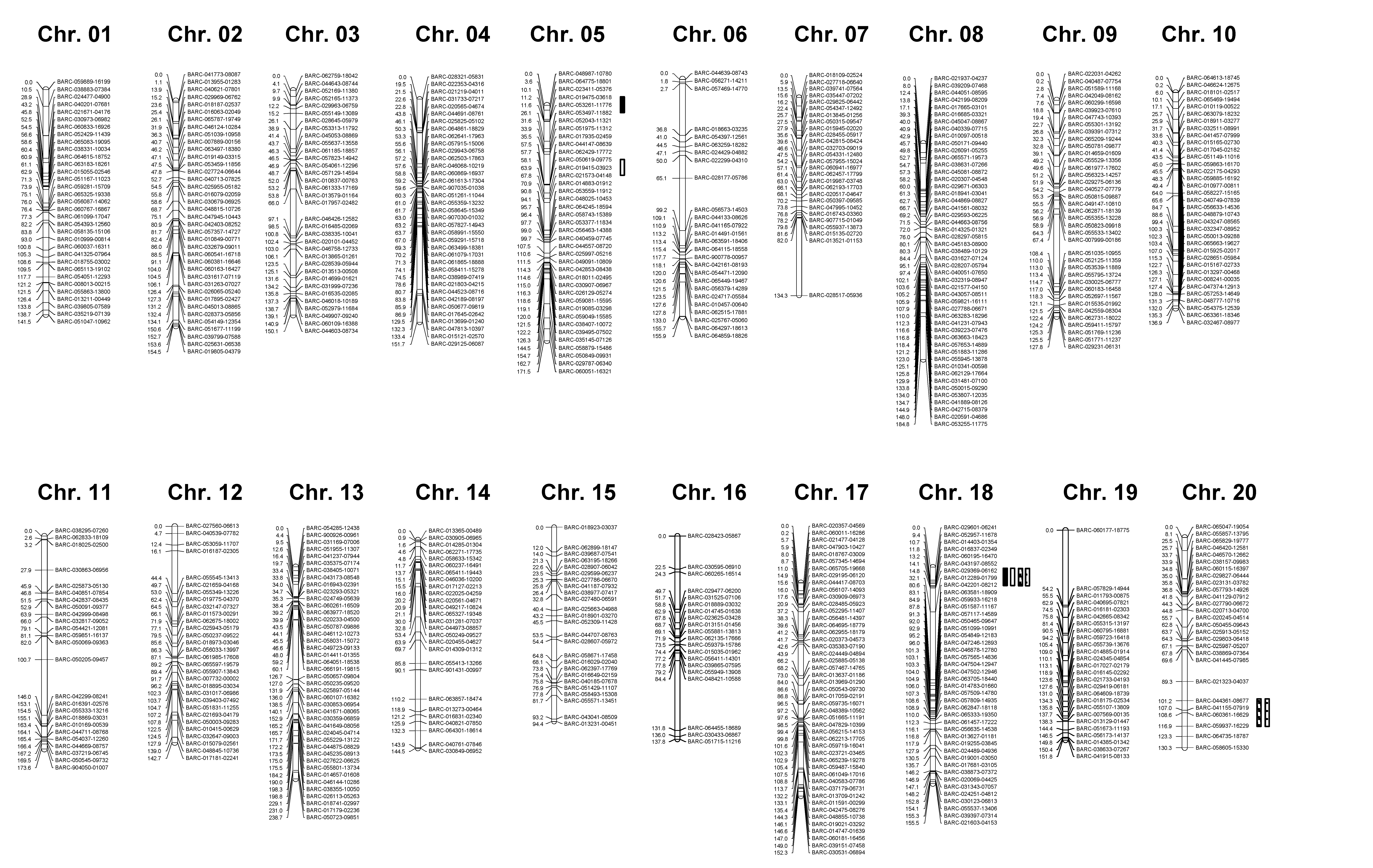

Supplement: Supplementary file 1 — Supplementary material 1 A genetic linkage map constructed using an F6:7 recombinant inbred line (RIL) population from a Hutcheson × PI 437655 cross. The confidence intervals of SCN resistance QTL were shown by the bars on the right of chromosomes. The bars filled with black color represent the QTL resistant to HG type 1.2.5.7 (PA2). The bars filled no color represent the QTL resistant to HG type 0 (PA3). The bars filled with slashes represent the QTL resistant to HG type 1.3.5.6.7 (PA14). The bars filled with black dots represent the QTL resistant to HG type 1.2.3.4.5.6.7 (LY2) (TIFF 2678 kb) [file 122_2014_2409_MOESM1_ESM.tif]

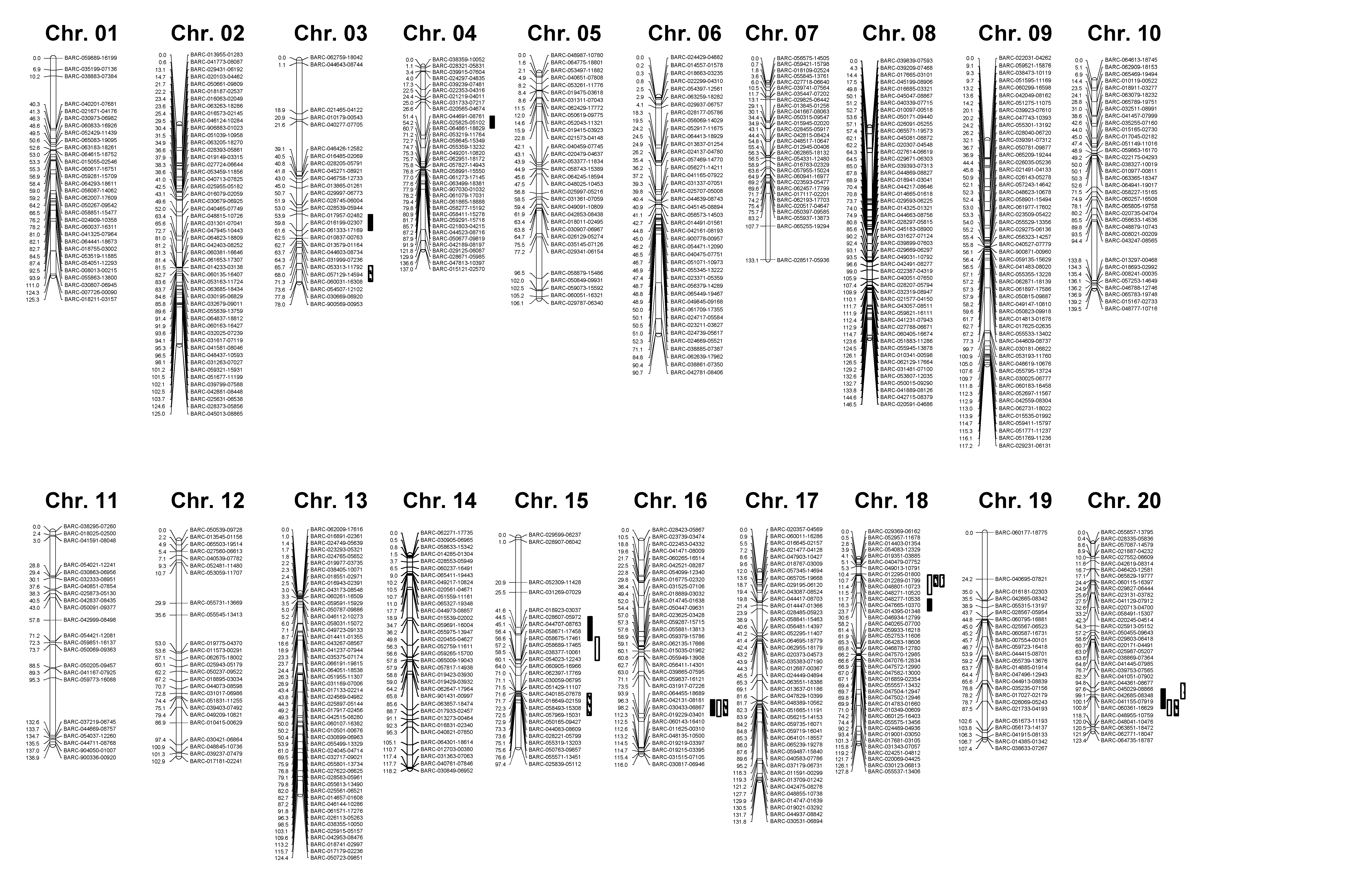

Supplement: Supplementary file 2 — Supplementary material 2 A genetic linkage map constructed using an F6:7 recombinant inbred line (RIL) population from a Williams 82 × PI 437655 cross. The confidence intervals of SCN resistance QTL were shown by the bars on the right of chromosome. The bars filled with black color represent the QTL resistant to HG type 1.2.5.7 (PA2). The bar filled no color represents the QTL resistant to HG type 0 (PA3). The bars filled with slashes represent the QTL resistant to HG type 1.3.5.6.7 (PA14). The bars filled with black dots represent the QTL resistant to HG type 1.2.3.4.5.6.7 (LY2) (TIFF 2817 kb) [file 122_2014_2409_MOESM2_ESM.tif]
